# (*E*)-Benzaldehyde *O*-{[3-(pyridin-3-yl)isoxazol-5-yl]meth­yl}oxime

**DOI:** 10.1107/S1600536812010732

**Published:** 2012-03-17

**Authors:** Rodolfo Moreno-Fuquen, Alix Elena Loaiza, John Diaz-Velandia, Alan R. Kennedy, Catriona A. Morrison

**Affiliations:** a Departamento de Química, Facultad de Ciencias, Universidad del Valle, Apartado 25360, Santiago de Cali, Colombia; b Laboratorio de Sintesis Orgánica, Facultad de Ciencias, Pontificia Universidad Javeriana, Bogota, DC, Colombia; c WestCHEM, Department of Pure and Applied Chemistry, University of Strathclyde, 295 Cathedral Street, Glasgow G1 1XL, Scotland

## Abstract

The asymmetric unit of the title compound, C_16_H_13_N_3_O_2_, contains two independent mol­ecules in which the pyridine and benzene rings form dihedral angles of 81.7 (2) and 79.8 (2)°, indicating the twist in the mol­ecules. In the crystal, weak C—H⋯N inter­actions link mol­ecules into chains along [100].

## Related literature

For organic synthesis of isoxazole systems, see: Giomi *et al.* (2008 ▶); Chukanov & Reznikov (2011 ▶). For the biological activity of isoxazole systems, see: Meyers *et al.* (2011 ▶); Basappa *et al.* (2003 ▶); Lee *et al.* (2009 ▶); Talley *et al.* (2000 ▶); Farrerons *et al.* (2003 ▶); Edgard *et al.* (2004 ▶); For hydrogen-bond graph-set motifs, see: Etter (1990 ▶). For hydrogen bonding, see: Nardelli (1995 ▶).
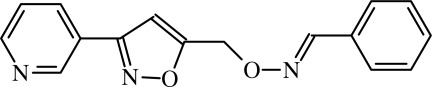


## Experimental

### 

#### Crystal data

C_16_H_13_N_3_O_2_
*M* *_r_* = 279.29
Orthorhombic, 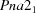

*a* = 19.364 (12) Å
*b* = 4.459 (3) Å
*c* = 31.775 (19) Å
*V* = 2744 (3) Å^3^
*Z* = 8
Mo *K*α radiation
μ = 0.09 mm^−1^
*T* = 100 K
0.40 × 0.01 × 0.01 mm

#### Data collection

Rigaku Saturn724+ diffractometer
17573 measured reflections
4762 independent reflections
3544 reflections with *I* > 2σ(*I*)
*R*_int_ = 0.086

#### Refinement

*R*[*F* ^2^ > 2σ(*F* ^2^)] = 0.059
*wR*(*F* ^2^) = 0.136
*S* = 0.99
4762 reflections
379 parameters
1 restraint
H-atom parameters constrained
Δρ_max_ = 0.32 e Å^−3^
Δρ_min_ = −0.25 e Å^−3^

### 

Data collection: *CrystalClear-SM Expert* (Rigaku, 2011 ▶); cell refinement: *CrystalClear-SM Expert*; data reduction: *CrystalClear-SM Expert*; program(s) used to solve structure: *SHELXS97* (Sheldrick, 2008 ▶); program(s) used to refine structure: *SHELXL97* (Sheldrick, 2008 ▶); molecular graphics: *ORTEP-3 for Windows* (Farrugia, 1997 ▶) and *Mercury* (Macrae *et al.*, 2006 ▶); software used to prepare material for publication: *WinGX* (Farrugia, 1999 ▶).

## Supplementary Material

Crystal structure: contains datablock(s) I, global. DOI: 10.1107/S1600536812010732/vm2159sup1.cif

Structure factors: contains datablock(s) I. DOI: 10.1107/S1600536812010732/vm2159Isup2.hkl

Supplementary material file. DOI: 10.1107/S1600536812010732/vm2159Isup3.cml

Additional supplementary materials:  crystallographic information; 3D view; checkCIF report

## Figures and Tables

**Table 1:** Hydrogen-bond geometry (Å, °)

*D*—H⋯*A*	*D*—H	H⋯*A*	*D*⋯*A*	*D*—H⋯*A*
C26—H26⋯N3^i^	0.95	2.43	3.374 (5)	174
C10—H10⋯N6^ii^	0.95	2.49	3.443 (5)	179

Symmetry codes: (i) 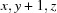; (ii) 
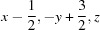
.

**Fig. 1.**
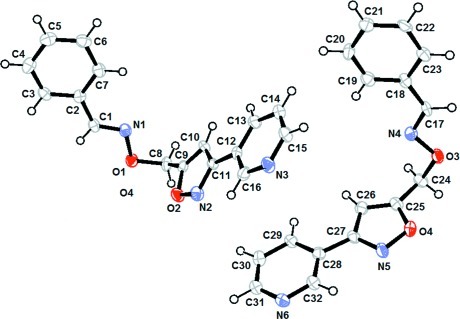
An ORTEP-3 (Farrugia, 1997) plot of (I) with displacement ellipsoids drawn at the 50% probability level. H atoms are shown as spheres of arbitrary radius.

**Fig. 2.**
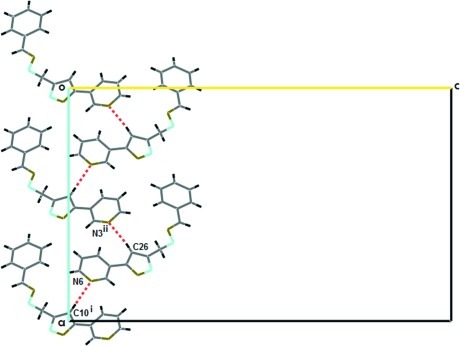
Part of the crystal structure of (I), showing the formation of chains along [100]. Symmetry code: (i) x - 1/2,-y + 3/2,z; (ii) x,y + 1,z.

## supplementary crystallographic information

### Comment

The isoxazoles are five-membered heterocyclic systems with one oxygen atom and one nitrogen atom at adjacent positions. These compounds are used as intermediates in organic synthesis due to their easy transformation into important groups such as enamino ketones, enoximes, 1,3-dicarbonyl compounds, γ-amino alcohols, and β-hydroxy nitriles (Giomi *et al.*, 2008; Chukanov & Reznikov, 2011).

They also have been widely used in the synthesis of nucleosides, alkaloids and other natural compounds. Many derivatives exhibit interesting applications in various fields such as agriculture, industry, and medicine. The wide spectrum of biological activities characteristic of these systems, comprises analgesic (Meyers *et al.*, 2011), antifungal (Basappa *et al.*, 2003), antiviral (Lee *et al.*, 2009), anti-inflammatory (Talley *et al.*, 2000), and antiobesity (Giomi *et al.*, 2008) activities.

In our research group, we are interested in the synthesis of nitrogen containing compounds with potential biological activity such as isoxazoles and oximes. The (*E*)-benzaldehyde *O*-(3-(pyridin-3-yl)isoxazol-5-yl)methyl oxime, (I), is an isoxazole analogue exhibiting important antibiotic (Farrerons *et al.*, 2003) and immunomodulator properties (Edgard *et al.*, 2004). On the other hand, the oxime function is an important pharmacophore group present in a wide variety of biologically active compounds, such as 3-oxiconazole and cefuroxime. Compound I was synthesized *via* 1,3-dipolar cycloaddition of an alkyne and a nitrile oxide obtained by treatment of (*E*)-nicotinaldehyde oxime with NaOCl. The reaction proceeded with high regioselectivity affording only the 5-substituted isomer in 45% yield. The molecular structure of I is shown in Fig. 1. The asymmetric unit of (I) contains two independent molecules (1) and (2). In both molecules, the isoxazole and pyridine rings are almost coplanar (r.m.s. deviation of all non-hydrogen atoms = 0.0044 Å). The dihedral angles between the mean planes defined by isoxazole and pyridine rings are 3.0 (3)° in molecule 1 and 5.8 (3)° in molecule 2. The pyridine and benzene rings form a dihedral angle of 81.7 (2)° in molecule 1 and 79.8 (2)° in molecule 2 indicating the twist in the molecules. The torsion angles of C8—O1—N1—C1 in (1) and C24—O3—N4—C17 in (2) are 176.2 (3) and -173.8 (3)° respectively and the least-squares fit of C2 C1 N1 O1 C8 C9 plane in (1) and C18 C17 N4 O3 C24 C25 plane in (2) show a r.m.s deviation of fitted atoms of 0.2117 and 0.2090 Å respectively, indicating the similar conformation of both molecules. The crystal packing is stabilized by weak C—H···N interactions (see Table 1, Nardelli, 1995). The molecules 1 and 2, are intertwined forming C(6) (Etter, 1990) chains of molecules along [100], see Fig. 2.

### Experimental

A stirred solution of (*E*)-benzaldehyde O-prop-2-ynyl oxime (318 mg, 2 mmol) and (E)- nicotinaldehyde oxime (122 mg, 1 mmol) in dichloromethane (4 ml) was placed on an ice bath during 5 minutes and then NaOCl in aqueous solution 5.25% (3 ml, 2.5 mmol) was added. The mixture was allowed to react for 30 minutes. After this period the phases were separated and the aqueous phase was extracted with AcOEt. The combined organic phases were dried with anhydrous Na_2_SO_4_, filtered and concentrated under low pressure. Purification of the crude mixture by flash column chromatography with 25% (*v*/*v*) AcOEt/hexane yielded a white solid (126 mg, 45% yield, mp 322 (1) *K*).

(*E*)-benzaldehyde *O*-(3-(pyridin-3-yl)isoxazol-5-yl)methyl oxime ^1^H NMR (300 MHz) δ, 9.04 (d, 1H), 8.70 (dd, 1H), 8.20 (ddd, 1H), 8.19 (s, 1H), 7.63–7.60 (m, 2H), 7.46–7.38 (m, 4H), 6.69 (t, 1H), 5.34 (d, 2H). ^13^C-NMR δ, 170.25, 159.10, 150.75, 150.45, 147.74, 134.31, 131.45, 130.37, 128.78, 127.30, 123.86, 101.24, 66.66. MS—EI *M*^+^ 279.1, 159.1 (100%).

### Refinement

The H-atoms were positioned geometrically [C—H= 0.95 Å for aromatic and C—H= 0.99 Å for methylene] and refined with *U*_iso_(H) 1.2 and 1.5 times *U*_eq_ of the parent atom, respectively.

### Crystal data

**Table:**

C_16_H_13_N_3_O_2_	*D*_x_ = 1.352 Mg m^−^^3^
*M**_r_* = 279.29	Melting point: 322(1) K
Orthorhombic, *P**n**a*2_1_	Mo *K*α radiation, λ = 0.71075 Å
Hall symbol: P 2c -2n	Cell parameters from 4687 reflections
*a* = 19.364 (12) Å	θ = 2.5–25.1°
*b* = 4.459 (3) Å	µ = 0.09 mm^−^^1^
*c* = 31.775 (19) Å	*T* = 100 K
*V* = 2744 (3) Å^3^	Needle, colourless
*Z* = 8	0.40 × 0.01 × 0.01 mm
*F*(000) = 1168	

### Data collection

**Table:**

Rigaku Saturn724+ diffractometer	3544 reflections with *I* > 2σ(*I*)
Radiation source: Rotating Anode	*R*_int_ = 0.086
Confocal monochromator	θ_max_ = 25.0°, θ_min_ = 3.8°
Detector resolution: 28.5714 pixels mm^-1^	*h* = −22→20
profile data from ω–scans	*k* = −4→5
17573 measured reflections	*l* = −37→37
4762 independent reflections	

### Refinement

**Table:**

Refinement on *F*^2^	Primary atom site location: structure-invariant direct methods
Least-squares matrix: full	Secondary atom site location: difference Fourier map
*R*[*F*^2^ > 2σ(*F*^2^)] = 0.059	Hydrogen site location: inferred from neighbouring sites
*wR*(*F*^2^) = 0.136	H-atom parameters constrained
*S* = 0.99	*w* = 1/[σ^2^(*F*_o_^2^) + (0.0521*P*)^2^] where *P* = (*F*_o_^2^ + 2*F*_c_^2^)/3
4762 reflections	(Δ/σ)_max_ < 0.001
379 parameters	Δρ_max_ = 0.32 e Å^−^^3^
1 restraint	Δρ_min_ = −0.25 e Å^−^^3^

### Special details

**Table:**

Geometry. All e.s.d.'s (except the e.s.d. in the dihedral angle between two l.s. planes) are estimated using the full covariance matrix. The cell e.s.d.'s are taken into account individually in the estimation of e.s.d.'s in distances, angles and torsion angles; correlations between e.s.d.'s in cell parameters are only used when they are defined by crystal symmetry. An approximate (isotropic) treatment of cell e.s.d.'s is used for estimating e.s.d.'s involving l.s. planes.
Refinement. Refinement of *F*^2^ against ALL reflections. The weighted *R*-factor *wR* and goodness of fit *S* are based on *F*^2^, conventional *R*-factors *R* are based on *F*, with *F* set to zero for negative *F*^2^. The threshold expression of *F*^2^ > σ(*F*^2^) is used only for calculating *R*-factors(gt) *etc*. and is not relevant to the choice of reflections for refinement. *R*-factors based on *F*^2^ are statistically about twice as large as those based on *F*, and *R*- factors based on ALL data will be even larger.

### Fractional atomic coordinates and isotropic or equivalent isotropic displacement parameters (Å^2^)

**Table:**

	*x*	*y*	*z*	*U*_iso_*/*U*_eq_	
O1	0.42469 (12)	1.0263 (6)	−0.10487 (8)	0.0291 (6)	
O2	0.54055 (14)	0.8039 (6)	−0.04845 (8)	0.0312 (7)	
O3	0.66365 (12)	1.0298 (6)	0.27422 (8)	0.0289 (6)	
O4	0.78145 (14)	0.8318 (6)	0.21677 (8)	0.0341 (7)	
N1	0.36765 (16)	0.8518 (7)	−0.08969 (10)	0.0272 (8)	
N2	0.56013 (16)	0.6236 (8)	−0.01381 (9)	0.0310 (8)	
N3	0.57737 (19)	0.2015 (7)	0.10467 (11)	0.0317 (8)	
N4	0.60742 (16)	0.8498 (7)	0.25889 (10)	0.0283 (8)	
N5	0.80334 (18)	0.6730 (8)	0.18076 (10)	0.0335 (9)	
N6	0.82896 (17)	0.3085 (9)	0.05894 (11)	0.0363 (9)	
C1	0.3367 (2)	0.7268 (9)	−0.12076 (13)	0.0251 (9)	
H1	0.3528	0.7638	−0.1485	0.030*	
C2	0.27783 (18)	0.5302 (8)	−0.11453 (11)	0.0236 (9)	
C3	0.25108 (19)	0.3751 (9)	−0.14916 (12)	0.0268 (9)	
H3	0.2716	0.4005	−0.1761	0.032*	
C4	0.1950 (2)	0.1849 (9)	−0.14465 (14)	0.0335 (10)	
H4	0.1774	0.0803	−0.1684	0.040*	
C5	0.1645 (2)	0.1470 (10)	−0.10568 (13)	0.0350 (10)	
H5	0.1262	0.0156	−0.1027	0.042*	
C6	0.1894 (2)	0.2993 (10)	−0.07096 (13)	0.0328 (11)	
H6	0.1679	0.2754	−0.0443	0.039*	
C7	0.2467 (2)	0.4896 (9)	−0.07537 (12)	0.0304 (9)	
H7	0.2644	0.5917	−0.0515	0.037*	
C8	0.4554 (2)	1.1802 (9)	−0.06927 (12)	0.0290 (10)	
H8A	0.4201	1.3090	−0.0558	0.035*	
H8B	0.4932	1.3113	−0.0794	0.035*	
C9	0.48330 (17)	0.9676 (8)	−0.03760 (11)	0.0222 (8)	
C10	0.4652 (2)	0.9032 (9)	0.00232 (11)	0.0282 (9)	
H10	0.4280	0.9851	0.0180	0.034*	
C11	0.51395 (19)	0.6865 (9)	0.01587 (12)	0.0226 (9)	
C12	0.51848 (18)	0.5373 (9)	0.05737 (12)	0.0261 (9)	
C13	0.47154 (19)	0.6129 (10)	0.08933 (12)	0.0303 (10)	
H13	0.4355	0.7529	0.0842	0.036*	
C14	0.47866 (19)	0.4803 (10)	0.12829 (13)	0.0345 (10)	
H14	0.4474	0.5266	0.1504	0.041*	
C15	0.5324 (2)	0.2773 (10)	0.13475 (13)	0.0347 (10)	
H15	0.5373	0.1889	0.1618	0.042*	
C16	0.5701 (2)	0.3278 (9)	0.06674 (12)	0.0294 (9)	
H16	0.6014	0.2725	0.0451	0.035*	
C17	0.5772 (2)	0.7288 (9)	0.29060 (12)	0.0248 (10)	
H17	0.5929	0.7721	0.3183	0.030*	
C18	0.51797 (18)	0.5220 (8)	0.28453 (11)	0.0240 (9)	
C19	0.4886 (2)	0.4702 (9)	0.24531 (12)	0.0306 (10)	
H19	0.5060	0.5698	0.2211	0.037*	
C20	0.4334 (2)	0.2713 (9)	0.24168 (13)	0.0311 (10)	
H20	0.4138	0.2329	0.2148	0.037*	
C21	0.40688 (19)	0.1297 (9)	0.27672 (12)	0.0318 (10)	
H21	0.3689	−0.0038	0.2740	0.038*	
C22	0.4360 (2)	0.1830 (10)	0.31618 (12)	0.0295 (10)	
H22	0.4181	0.0857	0.3404	0.035*	
C23	0.4910 (2)	0.3773 (9)	0.31983 (13)	0.0291 (9)	
H23	0.5109	0.4131	0.3467	0.035*	
C24	0.6926 (2)	1.1897 (9)	0.23951 (12)	0.0296 (10)	
H24A	0.7290	1.3259	0.2501	0.036*	
H24B	0.6561	1.3147	0.2265	0.036*	
C25	0.72282 (18)	0.9900 (9)	0.20662 (12)	0.0253 (9)	
C26	0.7059 (2)	0.9344 (9)	0.16578 (12)	0.0279 (9)	
H26	0.6675	1.0106	0.1505	0.034*	
C27	0.7574 (2)	0.7403 (9)	0.15124 (13)	0.0246 (9)	
C28	0.76501 (19)	0.6134 (9)	0.10804 (12)	0.0266 (9)	
C29	0.7201 (2)	0.7057 (10)	0.07652 (12)	0.0319 (10)	
H29	0.6832	0.8394	0.0824	0.038*	
C30	0.7309 (2)	0.5954 (10)	0.03557 (13)	0.0376 (10)	
H30	0.7012	0.6526	0.0132	0.045*	
C31	0.7852 (2)	0.4038 (11)	0.02849 (13)	0.0397 (11)	
H31	0.7924	0.3338	0.0006	0.048*	
C32	0.8173 (2)	0.4122 (10)	0.09790 (12)	0.0326 (10)	
H32	0.8465	0.3441	0.1199	0.039*	

### Atomic displacement parameters (Å^2^)

**Table:**

	*U*^11^	*U*^22^	*U*^33^	*U*^12^	*U*^13^	*U*^23^
O1	0.0330 (15)	0.0331 (17)	0.0211 (14)	−0.0047 (12)	−0.0017 (11)	0.0032 (12)
O2	0.0334 (16)	0.0400 (18)	0.0201 (16)	0.0057 (13)	0.0006 (13)	0.0018 (12)
O3	0.0346 (15)	0.0343 (18)	0.0179 (14)	−0.0090 (12)	0.0007 (11)	−0.0008 (12)
O4	0.0330 (17)	0.045 (2)	0.0239 (16)	−0.0007 (14)	−0.0060 (12)	−0.0055 (13)
N1	0.0316 (19)	0.028 (2)	0.0220 (18)	0.0003 (15)	−0.0010 (14)	0.0018 (14)
N2	0.033 (2)	0.041 (2)	0.0196 (18)	0.0037 (16)	−0.0030 (15)	0.0085 (15)
N3	0.036 (2)	0.038 (2)	0.0212 (19)	0.0052 (16)	−0.0026 (15)	0.0035 (16)
N4	0.0289 (18)	0.030 (2)	0.0260 (19)	−0.0003 (15)	0.0003 (15)	−0.0065 (16)
N5	0.034 (2)	0.046 (2)	0.0210 (18)	0.0077 (15)	−0.0015 (14)	−0.0038 (16)
N6	0.0274 (19)	0.053 (3)	0.029 (2)	0.0053 (16)	0.0036 (16)	−0.0066 (17)
C1	0.032 (2)	0.023 (2)	0.020 (2)	0.0047 (17)	−0.0016 (17)	0.0012 (16)
C2	0.027 (2)	0.020 (2)	0.024 (2)	0.0047 (16)	−0.0036 (16)	0.0051 (16)
C3	0.031 (2)	0.028 (2)	0.021 (2)	0.0078 (17)	0.0020 (17)	0.0032 (18)
C4	0.037 (3)	0.028 (3)	0.035 (3)	−0.0016 (19)	−0.010 (2)	0.004 (2)
C5	0.028 (2)	0.036 (3)	0.041 (3)	−0.0002 (18)	0.000 (2)	0.008 (2)
C6	0.029 (2)	0.039 (3)	0.031 (3)	0.006 (2)	0.003 (2)	0.0066 (19)
C7	0.037 (2)	0.031 (3)	0.023 (2)	0.0008 (18)	−0.0017 (17)	0.0052 (18)
C8	0.037 (2)	0.031 (3)	0.019 (2)	−0.0045 (19)	−0.0066 (17)	0.0017 (17)
C9	0.023 (2)	0.020 (2)	0.024 (2)	−0.0016 (16)	−0.0039 (16)	−0.0039 (15)
C10	0.028 (2)	0.039 (3)	0.018 (2)	0.0017 (18)	0.0031 (16)	−0.0046 (17)
C11	0.024 (2)	0.026 (2)	0.018 (2)	−0.0064 (16)	0.0026 (16)	0.0011 (16)
C12	0.025 (2)	0.032 (2)	0.0208 (19)	−0.0007 (17)	−0.0051 (16)	−0.0038 (17)
C13	0.029 (2)	0.038 (3)	0.024 (2)	0.0004 (18)	0.0002 (17)	0.0034 (18)
C14	0.033 (2)	0.046 (3)	0.024 (2)	−0.001 (2)	−0.0008 (18)	−0.0052 (18)
C15	0.041 (2)	0.044 (3)	0.019 (2)	−0.002 (2)	0.0017 (18)	0.0037 (18)
C16	0.026 (2)	0.035 (3)	0.027 (2)	−0.0025 (18)	−0.0054 (16)	0.0008 (18)
C17	0.036 (2)	0.025 (2)	0.014 (2)	0.0029 (17)	0.0004 (17)	−0.0042 (16)
C18	0.033 (2)	0.015 (2)	0.024 (2)	0.0027 (16)	0.0024 (17)	−0.0062 (15)
C19	0.033 (2)	0.033 (3)	0.025 (2)	0.0061 (19)	0.0018 (18)	0.0039 (18)
C20	0.035 (2)	0.034 (3)	0.025 (2)	−0.0041 (19)	−0.0078 (19)	−0.0029 (17)
C21	0.027 (2)	0.036 (3)	0.032 (2)	0.0031 (18)	0.003 (2)	−0.005 (2)
C22	0.027 (2)	0.040 (3)	0.022 (2)	0.0090 (18)	0.0093 (18)	0.0007 (18)
C23	0.034 (2)	0.029 (3)	0.024 (2)	0.0014 (18)	0.0052 (18)	−0.0033 (18)
C24	0.035 (2)	0.030 (2)	0.024 (2)	−0.0016 (18)	0.0057 (18)	0.0065 (18)
C25	0.031 (2)	0.021 (2)	0.024 (2)	−0.0023 (16)	0.0039 (16)	0.0039 (16)
C26	0.028 (2)	0.030 (2)	0.026 (2)	0.0018 (18)	−0.0011 (16)	0.0017 (17)
C27	0.028 (2)	0.025 (2)	0.020 (2)	−0.0051 (17)	0.0043 (17)	0.0035 (15)
C28	0.027 (2)	0.028 (2)	0.025 (2)	−0.0042 (17)	0.0024 (16)	0.0010 (17)
C29	0.029 (2)	0.044 (3)	0.023 (2)	0.0012 (19)	0.0037 (17)	−0.0007 (19)
C30	0.037 (2)	0.050 (3)	0.025 (2)	−0.002 (2)	−0.0027 (19)	−0.004 (2)
C31	0.033 (2)	0.062 (3)	0.025 (2)	−0.002 (2)	0.0015 (19)	−0.008 (2)
C32	0.032 (2)	0.042 (3)	0.023 (2)	−0.002 (2)	0.0023 (17)	0.0002 (19)

### Geometric parameters (Å, º)

**Table:**

O1—N1	1.435 (4)	C12—C16	1.400 (5)
O1—C8	1.451 (5)	C12—C13	1.404 (5)
O2—C9	1.371 (4)	C13—C14	1.379 (5)
O2—N2	1.415 (4)	C13—H13	0.9500
O3—C24	1.428 (5)	C14—C15	1.395 (6)
O3—N4	1.438 (4)	C14—H14	0.9500
O4—C25	1.375 (4)	C15—H15	0.9500
O4—N5	1.411 (4)	C16—H16	0.9500
N1—C1	1.282 (5)	C17—C18	1.484 (5)
N2—C11	1.330 (5)	C17—H17	0.9500
N3—C15	1.336 (5)	C18—C19	1.390 (5)
N3—C16	1.338 (5)	C18—C23	1.395 (5)
N4—C17	1.284 (5)	C19—C20	1.394 (6)
N5—C27	1.327 (5)	C19—H19	0.9500
N6—C32	1.341 (5)	C20—C21	1.379 (6)
N6—C31	1.355 (5)	C20—H20	0.9500
C1—C2	1.452 (5)	C21—C22	1.396 (5)
C1—H1	0.9500	C21—H21	0.9500
C2—C7	1.394 (5)	C22—C23	1.378 (6)
C2—C3	1.399 (5)	C22—H22	0.9500
C3—C4	1.386 (6)	C23—H23	0.9500
C3—H3	0.9500	C24—C25	1.493 (5)
C4—C5	1.382 (6)	C24—H24A	0.9900
C4—H4	0.9500	C24—H24B	0.9900
C5—C6	1.383 (6)	C25—C26	1.361 (5)
C5—H5	0.9500	C26—C27	1.400 (6)
C6—C7	1.404 (6)	C26—H26	0.9500
C6—H6	0.9500	C27—C28	1.492 (6)
C7—H7	0.9500	C28—C29	1.388 (5)
C8—C9	1.484 (5)	C28—C32	1.391 (6)
C8—H8A	0.9900	C29—C30	1.407 (5)
C8—H8B	0.9900	C29—H29	0.9500
C9—C10	1.347 (5)	C30—C31	1.373 (5)
C10—C11	1.417 (5)	C30—H30	0.9500
C10—H10	0.9500	C31—H31	0.9500
C11—C12	1.479 (5)	C32—H32	0.9500
			
N1—O1—C8	108.1 (3)	N3—C15—H15	118.5
C9—O2—N2	108.9 (3)	C14—C15—H15	118.5
C24—O3—N4	108.3 (3)	N3—C16—C12	123.2 (4)
C25—O4—N5	108.4 (3)	N3—C16—H16	118.4
C1—N1—O1	109.6 (3)	C12—C16—H16	118.4
C11—N2—O2	104.6 (3)	N4—C17—C18	120.8 (3)
C15—N3—C16	118.0 (4)	N4—C17—H17	119.6
C17—N4—O3	108.3 (3)	C18—C17—H17	119.6
C27—N5—O4	105.0 (3)	C19—C18—C23	119.4 (3)
C32—N6—C31	116.5 (4)	C19—C18—C17	122.4 (3)
N1—C1—C2	121.6 (4)	C23—C18—C17	118.2 (3)
N1—C1—H1	119.2	C18—C19—C20	119.6 (4)
C2—C1—H1	119.2	C18—C19—H19	120.2
C7—C2—C3	118.5 (3)	C20—C19—H19	120.2
C7—C2—C1	122.7 (4)	C21—C20—C19	120.7 (4)
C3—C2—C1	118.8 (3)	C21—C20—H20	119.7
C4—C3—C2	120.7 (4)	C19—C20—H20	119.7
C4—C3—H3	119.6	C20—C21—C22	119.8 (4)
C2—C3—H3	119.6	C20—C21—H21	120.1
C5—C4—C3	120.2 (4)	C22—C21—H21	120.1
C5—C4—H4	119.9	C23—C22—C21	119.7 (4)
C3—C4—H4	119.9	C23—C22—H22	120.2
C4—C5—C6	120.4 (4)	C21—C22—H22	120.2
C4—C5—H5	119.8	C22—C23—C18	120.8 (4)
C6—C5—H5	119.8	C22—C23—H23	119.6
C5—C6—C7	119.5 (4)	C18—C23—H23	119.6
C5—C6—H6	120.2	O3—C24—C25	113.4 (3)
C7—C6—H6	120.2	O3—C24—H24A	108.9
C2—C7—C6	120.6 (4)	C25—C24—H24A	108.9
C2—C7—H7	119.7	O3—C24—H24B	108.9
C6—C7—H7	119.7	C25—C24—H24B	108.9
O1—C8—C9	112.1 (3)	H24A—C24—H24B	107.7
O1—C8—H8A	109.2	C26—C25—O4	109.2 (3)
C9—C8—H8A	109.2	C26—C25—C24	132.9 (4)
O1—C8—H8B	109.2	O4—C25—C24	117.8 (3)
C9—C8—H8B	109.2	C25—C26—C27	104.8 (3)
H8A—C8—H8B	107.9	C25—C26—H26	127.6
C10—C9—O2	109.5 (3)	C27—C26—H26	127.6
C10—C9—C8	132.9 (4)	N5—C27—C26	112.6 (4)
O2—C9—C8	117.6 (3)	N5—C27—C28	119.9 (4)
C9—C10—C11	105.0 (3)	C26—C27—C28	127.5 (4)
C9—C10—H10	127.5	C29—C28—C32	118.7 (4)
C11—C10—H10	127.5	C29—C28—C27	119.3 (4)
N2—C11—C10	112.1 (3)	C32—C28—C27	122.0 (3)
N2—C11—C12	119.8 (3)	C28—C29—C30	118.0 (4)
C10—C11—C12	128.1 (3)	C28—C29—H29	121.0
C16—C12—C13	117.9 (4)	C30—C29—H29	121.0
C16—C12—C11	122.2 (3)	C31—C30—C29	118.9 (4)
C13—C12—C11	119.9 (3)	C31—C30—H30	120.5
C14—C13—C12	118.8 (4)	C29—C30—H30	120.5
C14—C13—H13	120.6	N6—C31—C30	123.8 (4)
C12—C13—H13	120.6	N6—C31—H31	118.1
C13—C14—C15	119.0 (4)	C30—C31—H31	118.1
C13—C14—H14	120.5	N6—C32—C28	124.0 (4)
C15—C14—H14	120.5	N6—C32—H32	118.0
N3—C15—C14	123.0 (4)	C28—C32—H32	118.0
			
C8—O1—N1—C1	176.2 (3)	C13—C12—C16—N3	−1.3 (6)
C9—O2—N2—C11	0.2 (4)	C11—C12—C16—N3	176.5 (4)
C24—O3—N4—C17	−173.8 (3)	O3—N4—C17—C18	−178.3 (3)
C25—O4—N5—C27	−0.4 (4)	N4—C17—C18—C19	−5.9 (6)
O1—N1—C1—C2	178.5 (3)	N4—C17—C18—C23	174.6 (4)
N1—C1—C2—C7	7.0 (6)	C23—C18—C19—C20	−1.0 (6)
N1—C1—C2—C3	−173.1 (3)	C17—C18—C19—C20	179.5 (4)
C7—C2—C3—C4	−0.1 (5)	C18—C19—C20—C21	1.2 (6)
C1—C2—C3—C4	179.9 (4)	C19—C20—C21—C22	−0.8 (6)
C2—C3—C4—C5	0.3 (6)	C20—C21—C22—C23	0.1 (6)
C3—C4—C5—C6	0.3 (6)	C21—C22—C23—C18	0.1 (6)
C4—C5—C6—C7	−1.0 (6)	C19—C18—C23—C22	0.4 (6)
C3—C2—C7—C6	−0.6 (5)	C17—C18—C23—C22	179.9 (4)
C1—C2—C7—C6	179.4 (4)	N4—O3—C24—C25	−62.8 (4)
C5—C6—C7—C2	1.1 (6)	N5—O4—C25—C26	1.0 (4)
N1—O1—C8—C9	62.2 (4)	N5—O4—C25—C24	−176.6 (3)
N2—O2—C9—C10	0.3 (4)	O3—C24—C25—C26	114.7 (5)
N2—O2—C9—C8	178.1 (3)	O3—C24—C25—O4	−68.4 (4)
O1—C8—C9—C10	−113.6 (5)	O4—C25—C26—C27	−1.1 (4)
O1—C8—C9—O2	69.3 (4)	C24—C25—C26—C27	175.9 (4)
O2—C9—C10—C11	−0.7 (4)	O4—N5—C27—C26	−0.3 (5)
C8—C9—C10—C11	−178.0 (4)	O4—N5—C27—C28	179.1 (3)
O2—N2—C11—C10	−0.6 (4)	C25—C26—C27—N5	0.9 (5)
O2—N2—C11—C12	−179.8 (3)	C25—C26—C27—C28	−178.5 (4)
C9—C10—C11—N2	0.8 (5)	N5—C27—C28—C29	−174.5 (4)
C9—C10—C11—C12	179.9 (4)	C26—C27—C28—C29	4.8 (6)
N2—C11—C12—C16	−0.4 (6)	N5—C27—C28—C32	3.3 (6)
C10—C11—C12—C16	−179.4 (4)	C26—C27—C28—C32	−177.4 (4)
N2—C11—C12—C13	177.3 (3)	C32—C28—C29—C30	−1.4 (6)
C10—C11—C12—C13	−1.7 (6)	C27—C28—C29—C30	176.4 (4)
C16—C12—C13—C14	0.5 (6)	C28—C29—C30—C31	−0.3 (6)
C11—C12—C13—C14	−177.2 (3)	C32—N6—C31—C30	−0.1 (7)
C12—C13—C14—C15	0.4 (6)	C29—C30—C31—N6	1.1 (7)
C16—N3—C15—C14	0.1 (6)	C31—N6—C32—C28	−1.8 (6)
C13—C14—C15—N3	−0.8 (6)	C29—C28—C32—N6	2.6 (6)
C15—N3—C16—C12	0.9 (6)	C27—C28—C32—N6	−175.2 (4)

### Hydrogen-bond geometry (Å, º)

**Table:**

*D*—H···*A*	*D*—H	H···*A*	*D*···*A*	*D*—H···*A*
C26—H26···N3^i^	0.95	2.43	3.374 (5)	174
C10—H10···N6^ii^	0.95	2.49	3.443 (5)	179

Symmetry codes: (i) *x*, *y*+1, *z*; (ii) *x*−1/2, −*y*+3/2, *z*.
